# Efficacy and safety of azvudine in symptomatic adult COVID-19 participants who are at increased risk of progressing to critical illness: a study protocol for a multicentre randomized double-blind placebo-controlled phase III trial

**DOI:** 10.1186/s13063-024-07914-3

**Published:** 2024-01-22

**Authors:** Xinlun Tian, Yan Xu, Luo Wang, Chongya Dong, Xiaoyan Yan, Junping Fan, Huaiya Xie, Hong Zhang, Jinglan Wang, Yongjian Liu, Yaqi Wang, Siqi Pan, Aohua Wu, Xueqi Liu, Chen Yao, Mengzhao Wang

**Affiliations:** 1 Department of Pulmonary and Critical Care Medicine, Peking Union Medical College Hospital, Chinese Academy of Medical Sciences & Peking Union Medical College, Beijing, 100730 China; 2 Peking University Clinical Research Institute, Beijing, China; 3 Department of Biostatistics, Peking University First Hospital, Beijing, China

**Keywords:** Azvudine, Disease progression, Mortality, Randomized controlled trial, Novel coronavirus infection

## Abstract

**Background:**

Severe acute respiratory syndrome coronavirus 2 will coexist with humans for a long time, and it is therefore important to develop effective treatments for coronavirus disease 2019 (COVID-19). Recent studies have demonstrated that antiviral therapy is a key factor in preventing patients from progressing to severe disease, even death. Effective and affordable antiviral medications are essential for disease treatment and are urgently needed. Azvudine, a nucleoside analogue, is a potential low-cost candidate with few drug interactions. However, validation of high-quality clinical studies is still limited.

**Methods:**

This is a multicentre, randomized, double-blind, placebo-controlled phase III clinical trial involving 1096 adult patients with mild-to-moderate symptoms of COVID-19 who are at high risk for progression to severe COVID-19. Patients will be randomized to (1) receive azvudine tablets 5 mg daily for a maximum of 7 days or (2) receive placebo five tablets daily. All participants will be permitted to use a standard treatment strategy except antiviral therapy beyond the investigational medications. The primary outcome will be the ratio of COVID-19-related critical illness and all-cause mortality among the two groups within 28 days.

**Discussion:**

The purpose of this clinical trial is to determine whether azvudine can prevent patients at risk of severe disease from progressing to critical illness and death, and the results will identify whether azvudine is an effective and affordable antiviral treatment option for COVID-19.

**Trial registration:**

ClinicalTrials.gov NCT05689034. Registered on 18 January 2023.

**Supplementary Information:**

The online version contains supplementary material available at 10.1186/s13063-024-07914-3.

## Introduction

### Background and rationale {6a}

Since December 2019, coronavirus disease 2019 (COVID-19) caused by severe acute respiratory syndrome coronavirus 2 (SARS-CoV-2) has become a global pandemic. By Nov 8, 2023, according to data from the World Health Organization (WHO), 771,820,937 cases of COVID-19 had been confirmed globally, and 6,978,175 of the patients had died (http://covid19.who.int) [1]. The disease continues to spread globally and has become a major threat to human health, bringing an enormous socioeconomic burden to individuals, families, and society [2]. Multiple society guidelines recommend that COVID-19 patients be treated with antiviral medication, corticosteroids, and biological agents according to the disease severity and clinical manifestations [3, 4]. Among these treatments, antiviral therapy is a key factor in preventing patients from progressing to severe disease, viral sepsis, and even death. 3-Chymotrypsin-like cysteine protease enzyme inhibitors (such as nirmatrelvir-ritonavir, known as Paxlovid) have been proven to be effective in reducing mortality in patients with a risk of disease progression to severe COVID-19 in both double-blind, randomized, controlled studies and the real world [5, 6]. In addition, RNA-dependent RNA polymerase (RdRp) nucleoside analogues, such as molnupiravir and remdesivir, have also showed evidence of accelerating SARS-CoV-2 RNA clearance or reducing hospitalization or death in COVID-19 patients [7–10]. If applied early, the viral load can decline over time, thus reducing the physiological damage directly caused by coronavirus and mitigating the inflammatory storm [11, 12]. Due to the high cost of the antiviral medications available on the market, effective and more affordable medications are still essential for disease treatment and are urgently needed.

SARS-CoV-2 is a new type of RNA virus. Azvudine is an RdRps nucleoside analogue given as 5 mg once daily for up to 14 days that has gained emergency approval for treatment of COVID-19 patients in the diagnosis and treatment protocol for COVID-19 in China (trial version 9) since August 2022 [13, 14]. However, the drug has not been validated by high-quality clinical studies. Compared with Paxlovid, a widely used anti-COVID-19 drug combination, azvudine is advantageous because of its lower price and fewer drug interactions. Quite a few retrospective studies have also found a decreased mortality among users of azvudine [15–17]. Shen et al. reported that azvudine could reduce mortality in COVID-19 patients (*HR*: 0.26; 95% *CI*: 0.07–0.94), by comparing 226 COVID-19 patients treated with azvudine with 226 matched controls. Another study conducted by Zong et al. showed that azvudine was associated with reduced in-hospital mortality through propensity score matching and univariate analysis (*OR* 0.38, 95% *CI* 0.23–0.62). A multicentre study including 1110 hospitalized patients also demonstrated the effectiveness of azvudine in reducing mortality in COVID-19 patients by multivariate analysis and inverse probability weighting (*OR* 0.75, 95% *CI* 0.61–0.93). However, well-designed, large-scale randomized trials focusing on critical illness and hospitalization prevention are lacking.

This multicentre, randomized, double-blind, placebo-controlled, phase III study aimed to determine whether azvudine can prevent patients with mild-to-moderate symptoms of COVID-19 at risk of severe disease from progressing to critical illness and death.

### Objectives {7}

The objectives of this trial were to assess the efficacy and safety of azvudine for COVID-19 patients with risk factors for progressing to critical illness. We hypothesized that azvudine would be an effective and safe antiviral drug for COVID-19 patients.

### Trial design {8}

This is a prospective multicentre, placebo-controlled, randomized, double-blind phase III trial, with a superiority design. A total of 1096 symptomatic adult patients with COVID-19 infection who are at increased risk of progressing to critical illness, based on their demographics and comorbidities, will be recruited from January 2023 through January 2024 [5]. The patients will be randomized at a 1:1 ratio to the treatment group and placebo group and treated with azvudine and placebo, respectively (Additional file 1). The investigator will observe and record subjects’ disease progression or remission and adverse drug reactions and analyse the data. Double blinding will be used in the trial.

This protocol was designed according to the Standard Protocol Items: Recommendations for Interventional Trials (SPIRIT) 2013 Statement (Additional file 2) [18] and registered at ClinicalTrials.gov (ID: NCT05689034).

## Methods: participants, interventions, and outcomes

### Study setting {9}

The Peking Union Medical College Hospital (PUMCH) is the initiator and responsible for this programme. Several hospitals in China participate as subcentres for patient recruitment, diagnosis and treatment, and data collection. The subcentres are listed in the China Human Genetic Resources Service System (https://apply.hgrg.net) and the Medical Research Registration and Filing Information System (https://www.medicalresearch.org.cn/login).

### Eligibility criteria {10}

#### Inclusion criteria

1. Age of 18 years or older (inclusive)
2. No more than 5 days since symptom onset and a positive result of nucleic acid and/or antigen test for COVID-19 within 5 days before enrolment
3. The presence of at least one symptom related to COVID-19 infection at the time of enrolment (Table 1)

**Table 1:** Subject’s symptom recording form

**Symptoms**	D1	D2	D3	D4	D5	D6	D7	……	D14	D28
Cough, sputum										
Sore throat										
Stuffy nose										
Running nose, sneezing										
Decreased sense of smell										
Decreased sense of taste										
Dyspnoea										
Headache										
Fever, chills										
Highest temperature of the day										
NSAIDs or not										
Muscle or joint pain										
Fatigue										
Nausea										
Vomiting										
Diarrhoea										
Adverse events
4. Consent to use effective pregnancy control measures for females of child-bearing age and a negative urine pregnancy test
5. The presence of at least one of the following characteristics associated with an increased risk of developing severe illness:
  Age ≥ 65 years
  Body mass index > 30 kg/m.^2^
  Current smokers (still smoking within 30 days before enrolment and smoking at least 100 cigarettes in total) [5]
  Status of immunosuppression, including but not limited to the following:
    Haematopoietic stem cell transplantation or solid organ transplantation
    Primary immunodeficiency disease
    Prolonged use of corticosteroids (≥ 20 mg/day for at least 14 days in the case of prednisone within the last 30 days)
    Use of biologic agents (such as infliximab)
    Use of immune-modulators (including but not limited to methotrexate, azathioprine)
    Chemotherapy and/or radiation therapy for any malignancies within 90 days (for chest radiation therapy, the time interval should be more than 6 months).
  Chronic lung disease (asthma under treatment, bronchiectasis, chronic obstructive pulmonary disease, pulmonary hypertension, obstructive sleep apnoea syndrome, interstitial lung disease, etc.)
  Hypertension
  Cardiovascular and cerebrovascular diseases (myocardial infarction, stroke, transient ischaemic attack, heart failure, angina pectoris requiring nitrate therapy, coronary artery bypass grafting, post-percutaneous coronary intervention, post carotid endarterectomy, and aortic bypass surgery, etc.)
  Type 1 or type 2 diabetes
  Neurodevelopmental abnormalities (such as cerebral palsy, Down’s syndrome) or other genetic or metabolic syndromes and severe congenital malformations
  Active tumours (excluding localized skin cancer)
  No vaccination against COVID-19

### Exclusion criteria

1. Known or suspected allergy to the components of azvudine tablets
2. Patients diagnosed with severe or critical COVID-19 infection on screening
3. Patients with severe liver disease (total bilirubin ≥ 2 × upper limit of normal [ULN], alanine aminotransferase [ALT], and aspartate aminotransferase [AST] ≥ 3 × ULN)
4. Patients with severe renal insufficiency (glomerular filtration rate ≤ 60 mL/min/1.73 m^2^) or currently on continuous renal replacement therapy, haemodialysis, and/or peritoneal dialysis
5. Patients with malabsorption syndrome or any other condition compromising gastrointestinal absorption or patients requiring parenteral nutrition or with difficulty taking the investigational product orally
6. Patients with known HIV infection
7. Patients with diabetic ketosis or a hyperosmolar hyperglycaemic state
8. Patients with a total neutrophil count < 750 cells/L
9. Pregnant or lactating women or those who plan to have a child during participation in this study and within 6 months after the end of this study
10. Patients currently participating in another interventional clinical trial or who are currently using another investigational product
11. Patients with other uncontrolled active infections (must be aetiologically confirmed) in addition to COVID-19 infection
12. The presence of any comorbidities requiring hospitalization and/or a surgical procedure within 7 days prior to the start of this study or a comorbidity considered life-threatening within 30 days prior to the start of this study, as determined by the investigator
13. Patients who have received or are expected to receive convalescent plasma for COVID-19
14. Patients previously treated with antiviral agents that have been proven to be effective against COVID-19, including but not limited to nirmatrelvir/ritonavir or molnupiravir
15. Patients with other conditions that make it inappropriate for the participant to take part in this trial at the investigator’s discretion

The risk factors for critical infection described in the inclusion criteria and the adverse drug reactions and patient comorbidities that may affect clinical outcomes described in the exclusion criteria were derived from reported studies [19, 20].

### Who will take informed consent? {26a}

The informed consent will be obtained from COVID-19 patients who meet all inclusion criteria and does not meet any exclusion criteria.

Before being enrolled in the trial, a researcher who has received training will provide patients and their families with an introduction to the study’s purpose and content. The potential benefits and risks will also be explained to them. A 24-h timeframe will be given to the patients and their families to decide whether to participate in the trial, ensuring that their participation is completely voluntary. Additionally, it will be clarified to the patients that their decision to participate or not will have no impact on their regular treatment. Any identifying information, such as names and hospital numbers, will be encoded to maintain confidentiality. The personal information of all participants will always be kept private.

### Additional consent provisions for collection and use of participant data and biological specimens {26b}

Not applicable. No data and specimens will be used for other studies.

## Interventions

### Explanation for the choice of comparators {6b}

This is a prospective placebo-controlled, randomized, double-blind phase III trial. The comparator is placebo five tablets once daily for 7 days. The placebos are manufactured and packaged through the same process as the study drug and are produced by the same manufacturer and product line. The placebo formulation provided has the same physical appearance as the active formulation, contains the same inactive ingredients, and will be used in the study for masking purposes.

### Intervention description {11a}

For the purposes of this protocol, the study intervention refers to azvudine 5 mg (five tablets once daily) and a matching placebo (five tablets once daily) for seven continuous days (Table 2). The medication and placebo are provided by Henan Genuine Biotech Co., Ltd. (Pingdingshan, China) and produced by designated, established manufacturers. All participants will be permitted to use a standard treatment strategy except antiviral therapy beyond the investigational medications.

**Table 2:** Arms and interventions

Arm	No. of participants	Interventions
Placebo group	548	Azvudine po. 5 mg once daily for 7 days + COVID-19 treatment regimens other than antiviral medications^a^
Treatment group	548	Placebo po. five tablets once daily for 7 days + COVID-19 treatment regimens other than antiviral medications^a^

^a^COVID-19 treatment regimens other than antiviral medications, including but not limited to oxygen therapy, respiratory support, symptomatic medications, glucocorticosteroids, and biologic agents such as interleukin-6 receptor inhibitors and tyrosine kinase inhibitors. Among other things, the programme does not restrict the use of nonsteroidal anti-inflammatory drugs to avoid adding additional discomfort to the subjects

### Criteria for discontinuing or modifying allocated interventions {11b}

#### Participants will withdraw from the study for any of the following reasons:

1. Participants with poor compliance who cannot cooperate with the clinical examination and follow-up.
2. Patients who voluntarily quit this clinical trial.

#### Patients will be advised to discontinue medication under the following conditions:

1. The illness of the subject worsens, and the researcher determines that the benefit of the subject is poor and the subject is unwilling to continue receiving trial drug treatment.
2. The subject has experienced serious adverse events (SAEs) related to the investigational drug (definitely related, likely related, possibly related), and the researcher has determined that it is not suitable to continue participating in this trial.
3. Other conditions occur for which the researcher believes that the subject is not suitable to continue receiving investigational drug. Serious unexpected suspected adverse reactions must be reported and documented according to the appropriate requirements of the research centre’s institutional or ethics committee.

### Strategies to improve adherence to interventions {11c}

The clinical research coordinator will determine if subjects are recording their condition daily by checking the subject’s symptom recording form (Table 1) and reminding each subject to take their medication daily. Upon completion of their medication, all subjects will be expected to return the medication package upon request.

### Relevant concomitant care permitted or prohibited during the trial {11d}

Relevant concomitant care was shown in Table 2.

### Provisions for posttrial care {30}

During outpatient treatment or after discharge from the hospital, study subjects should follow the following instructions and abide by the precautions specified by the research centre: continue rehabilitation for 3 to 6 weeks, wear a mask if necessary, maintain hand hygiene and do not share personal belongings, pay special attention to avoiding contact with high-risk groups, and report immediately to the principal investigator or his or her team in the event of fever and/or respiratory symptoms.

All subjects will be followed up for the disease through monitoring, consultations, and examinations that will benefit the patient’s treatment and recovery. If the patient’s condition warrants, additional outpatient consultation or hospitalization will be arranged for the subject in addition to routine visits.

### Outcomes {12}

The primary outcome of this trial is the ratio of COVID-19-related critical illness and all-cause mortality among the two groups within 28 days. COVID-19-related critical illness is defined as a patient who requires mechanical ventilation or extracorporeal membrane oxygenation or a patient maintaining oxygen saturation (SpO_2_) above 90% who requires high-flow oxygen inhalation with a fraction of inspired oxygen (FiO_2_) not less than 60% or requires noninvasive ventilation with a FiO_2_ not less than 60%.

The secondary outcomes are as follows: (1) COVID-19 viral load measured from pharyngeal swabs at the D1, D3, D7, and D14 timepoints, (2) incidence of azvudine-related adverse events (AEs) and serious adverse events, (3) duration of COVID-19-related symptoms, (4) all-cause mortality within 6 months, (5) days of hospitalization and intensive care unit stay, and (6) the ratio of COVID-19-related severe illness defined on the China Formula of Diagnosis and Treatment for COVID-19 (trial version 10). In detail are as follows: (1) shortness of breath with respiratory rate ≥ 30 breaths/min, (2) SpO_2_ ≤ 93% when inhaling air at rest, (3) partial pressure of arterial oxygen/FiO_2_ ≤ 300 mmHg, and (4) progressive worsening of clinical symptoms and obvious lesion progression > 50% on chest imaging within 24 to 48 h.

The safety endpoints consist of adverse events that emerge during or after the treatment period and subsequent observation period until 28 days. SAEs and AEs leading to discontinuation of the trial drug or placebo will be coded according to the definition of severe adverse events published by the FDA (https://www.fda.gov/safety/reporting-serious-problems-fda/what-serious-adverse-event).

### Participant timeline {13}

After randomization, patients will receive the allocated interventions for 7 continuous days and be followed up for 6 months. The study schedule includes a screening visit; four treatment visits on day 1, 3, 7, and 14; and three follow-up visits on day 28, 3, months and 6 months. We will collect the patients’ survival status at follow-up visits. The participant timeline is shown in Table 3.

**Table 3:** Schedule of screening, intervention and follow-up of the trial

Study periods	Screening	Treatment periods						Follow-up periods		
Time line		d1	d3 + / − 1	d4 to d6^a^	d7 + / − 1	d8 to d13^a^	d14 + / − 2	D28^a^ + / − 3	3 M^a^ + / − 7	6 M^a^ + / − 7
Consent form	X									
Check with inclusion and exclusion criteria	X									
Demographic characteristics	X									
Symptoms	X	X	X	X	X	X	X	X	X	X
Vital sign	X	X	X		X		X			
Adverse event		X	X	X	X	X	X			
Viral load		X	X		X		X			
Chest image (DXR or CT)		X			X		X	X		
Pregnancy test	X						X			
Blood routine	X	X	X		X		X			
Ferritin		X								
Interleukin-6		X								
Hypersensitive C-reactive protein		X	X		X		X			
Blood biochemical test	X	X	X		X		X			
Coagulation		X			X					
Oxygen level	X									
Cardiac troponin and N-terminal pro-brain natriuretic peptide		X			X					
Concomitant medication		X								

^a^Visits by phone calls

### Sample size {14}

This study aims to determine the efficacy of a new antiviral compared to placebo in reducing critical illness or all-cause mortality on day 28 in COVID-19 patients.

The sample size was calculated based on the superiority test. The primary endpoints are the disease critical rate and mortality rate. Based on the data reported in Hong Kong for patients with COVID-19 from the end of 2020 to March 2022, the number of deaths and serious illnesses was 6866 and 8875, respectively [21]. Previous data on another antiviral drug, Paxlovid, showed that the placebo group had 65 hospitalizations and 12 deaths, while the Paxlovid group had no deaths and 8 hospitalizations [5]. Combined with data from Hong Kong, it was speculated that there may be 15–16 severe patients in the placebo group and 2 severe patients in the Paxlovid group. Therefore, the disease critical rate and mortality rate in other countries and China are assumed to be 2.6% in the standard treatment group and can be reduced to 0.19% with effective treatment. With a power of 90% (1 sided *α* = 0.025 and *β* = 0.10), a sample size of 986 patients in each group is needed. The sample size calculations were performed using SAS software, version 9.4 (SAS Institute Inc., USA).

### Recruitment {15}

To account for a 10% dropout rate, the sample size increased to 1096 participants (548 in the azvudine group and 548 in the control group). To enrol enough participants, we set up special screening staffs in the fever clinic and emergency department where COVID-19 patients often visit, and screening staffs distribute COVID-19 antigen to suspected fever patients to identify candidates. In order to complete such large amount patients’ selection as soon as possible, we selected 46 study sites localized in 17 different provinces in China Mainland; thus, we will obtain timely enrolment to the greatest extent possible.

## Assignment of interventions: allocation

### Sequence generation {16a}

Eligible patients will be randomized in a 1:1 ratio to receive azvudine tablets or placebo only and receive the standard treatment strategy immediately after signing the informed consent form. The permutated-block randomization sequence is generated by computer, and randomization will be performed on a web-based randomization system.

### Concealment mechanism {16b}

In the concealment of the randomization sequence, the block size will be ensured by the web-based randomization system.

### Implementation {16c}

The staff of the central random system will generate the randomization sequence. The intervention will be decided by the randomization system, and the researcher will be blind to the treatment allocation plan.

## Assignment of interventions: blinding

### Who will be blinded {17a}

This was a double-blinded study, which means that researchers and patients will be blinded to the study intervention allocation.

The blinding will be ensured by producing and packaging (the study drug and the placebo cannot be differentiated by their appearances and packages).

A qualified staff member will dispense the study intervention using the IRT system via unique container numbers in the bottles and blister cards provided. Another staff member will verify the dispensing the blinded medication. The participant should be instructed to maintain the product in the bottle and blister cards, as appropriate, provided throughout the course of dosing and return the bottle and blister cards to the site at the next study visit. The study intervention will be administered in a blinded fashion to the participants.

### Procedure for unblinding if needed {17b}

The Interactive Response Technology (IRT) system will be programmed with instructions to maintain blinding. For anticipated and unanticipated serious adverse events, emergency unblinding may be performed only if the investigator must know the treatment grouping information to proceed in the event of an emergency in the subject (e.g. requiring resuscitation). The safety of the participant will always be the primary consideration in making this decision. The reason for unblinding and the date it occurs must be documented in the source documentation and case report form (CRF). The study-specific IRT reference manual and investigational product manual will provide contact information and further details on how to use the IRT system.

Unless efficacy is demonstrated at an interim analysis, the study will be unblinded after the statisticians finalize the statistical plan and the audit report in the data plan and lock the database. Details of the unblinded sponsor staff supporting the independent data monitoring committee (IDMC) and the timing of unblinding will be outlined in the unblinding plan.

## Data collection and management

### Plans for assessment and collection of outcomes {18a}

During the enrolment process, we plan to collect demographic information, including age, height, weight, blood pressure, heart rate, past medical history, smoking history, and laboratory parameters such as leukocytes, neutrophils, creatinine, uric acid, myocardial enzymes, pro-brain natriuretic peptide, hypersensitive C-reactive protein, D-dimer, and viral load upon admission (Table 3). These data are derived from the electronic medical record system of each participating hospital and will be documented on an electronic case report form (eCRF, https://study.cims-medtech.com/CIMS_V5/PlatFrame.aspx). Any AEs, including clinical symptoms and changes in vital signs, such as headache, dizziness, and nausea, and abnormalities observed in laboratory examination, including elevated serum creatinine AST or ALT, will also be documented. The correlation of AEs and the study drug or placebo will be carefully investigated. Furthermore, any changes in the patients’ conditions will be recorded during the follow-up period, which includes the 28th day after admission and up to 6 months.

Patients who are admitted to the hospital will be monitored on a daily basis until either a primary endpoint event occurs or they are discharged. A telephone follow-up will be conducted on the 28th day after admission to gather information about the patients’ survival status. For patients who remain hospitalized beyond 28 days, endpoint events will be recorded on the 30th day of their hospital stay.

### Plans to promote participant retention and complete follow-up {18b}

Laboratory tests at the follow-up visit are all necessary to observe condition alterations or to ensure the safety of the patient. The current study showed that nucleic acid conversion was delayed compared to viral culture and was highest at 14 days, so we collected nucleic acid samples again at day 14 [22].

### Data management {19}

This project is adopting electronic data management, and the relevant information of patients will be recorded in the electronic medical record system and entered onto the eCRF. Data management will be carried out through an electronic data capture system.

### Confidentiality {27}

All datasets will be stored on the research platform of the Clinical Information Management Suite and password-protected. The principal investigators have direct access to all datasets through this research platform. To ensure confidentiality, data distributed to project team members will be kept confidential from any information identifying participants.

### Plans for collection, laboratory evaluation, and storage of biological specimens for genetic or molecular analysis in this trial/future use {33}

It is not applicable. All collected specimens will be used to evaluate condition alteration and safety of intervention and will not be used for other studies. We did not collect and use of participant data and biological specimens in the current trial, and for future use, additional consent provisions are not necessary for our protocol.

## Statistical methods

### Statistical methods for primary and secondary outcomes {20a}

All data will be analysed using SAS software, version 9.4 (SAS Institute Inc., USA). A two-tailed *p* < 0.05 will be considered statistically significant in all statistical tests (except for the main efficacy indicators and other specified instructions).

The full analysis set (FAS), modified intention-to-treat set (mITT), per-protocol set (PPS), and safety set (SS) will be divided for analysis. FAS includes all eligible and randomized patients. PPS includes eligible patients with better compliance (80–120%), and completing necessary items of CRF.SS includes all patients enrolled in the study who have received at least one dose of medication and have at least one safety evaluation. Baseline characteristics analysis will be based on FAS, efficacy analysis will be based on FAS and PPS, and safety analysis will be based on SS. Unblinded patients will be excluded from PPS.

Continuous data will be summarized as the mean ± standard deviation, median, minimum, maximum, lower quartile, and upper quartile. Categorical data will be presented and summarized as frequencies and percentages in each category.

Between-group comparisons will be conducted by using paired *t*-tests or Wilcoxon rank tests for continuous data, chi-square tests or Fisher exact tests for categorical data, Wilcoxon rank tests or CMH tests for ranked data, and Kaplan‒Meier estimates for time-to-event data. The principles of intention-to-treat analysis will be used.

Efficacy analysis will be based on FAS and PPS. The primary efficacy endpoints are COVID-19-related critical illness and all-cause mortality within 28 days (whichever happens first), which will be analysed based on a time-to-event manner. Kaplan‒Meier method will be used to describe the incidence rates of the primary endpoint in each group separately at 28 days. Between-group comparisons will be performed by the log rank test. A Cox regression model will be used to estimate the hazard ratio and corresponding 95% confidence interval and adjust for potential covariates such as centres. Secondary efficacy endpoint analysis will be performed according to general statistical considerations. The specifics of these methods will be thoroughly outlined in the statistical analysis plan.

### Interim analyses {21b}

An interim analysis is planned to assess both efficacy and futility, and a sample size re-estimation will be conducted once the 548th participants in the mITT analysis set complete the day 28 assessments. The IDMC, supported by an unblinded statistical centre, will regularly review safety data and evaluate the interim efficacy analysis.

### Methods for additional analyses (e.g. subgroup analyses) {20b}

No additional analyses will be performed.

### Methods in analysis to handle protocol nonadherence and any statistical methods to handle missing data {20c}

The primary endpoint is defined as time-to-event data, non-endpoint events will be defined as censoring events, and the censoring date will be set as the last follow-up date. No carry-forward or multiple imputations will be conducted for missing data.

Missing data of secondary endpoints or safety endpoints will not be imputed. Missing data due to intercurrent events will be imputed by a multiple imputation approach under a prespecified estimand.

### Plans to give access to the full protocol, participant-level data, and statistical code {31c}

The datasets used and/or analysed during the current study will be available from the corresponding author upon reasonable request.

## Oversight and monitoring

### Composition of the coordinating centre and trial steering committee {5d}

This study was initiated by PUMCH and involves multiple centres. There is no other coordinating centre. Investigators from PUMCH act as coordinating investigators responsible for liaising and coordinating with other hospitals involved in the trial to promote and monitor the progress of the trial, with the assistance of the Contract Research Organization and Site Management Organization. The trial steering committee consists of the PUMCH clinical research team. The study team meets weekly to assess progress and data analysis. Meetings with subcentre investigators are held as needed to oversee the progress of the trial and to provide advice on problems encountered in subcentre project implementation.

The roles and responsibilities are as follows: the coordinating centre (Universidade Federal de Ciências da Saúde de Porto Alegre) is responsible for overseeing the overall progress of the trial, ensuring adherence to protocols, and liaising with involved universities.

### Composition of the data monitoring committee, its role, and reporting structure {21a}

The Data and Safety Monitoring Board (DSMB) of this clinical trial was established on June 18, 2023. The primary responsibility of the DSMB is to provide an independent review and evaluation of safety data in accordance with the protocol. It is responsible for reviewing safety results in a blinded or unblinded fashion, assessing adverse events collected in the study, evaluating cumulative SAE reports, and, based on the results of the cumulative analysis of the data obtained, providing recommendations to the sponsor regarding trial execution and study adjustments to further safeguard the interests and safety of the subjects and to ensure that these potential risks are controlled in a timely and effective manner. The DSMB consists of five people, including the DSMB chairperson, members, and secretary, who are independent of the sponsor and competing interests. DSMB members and statisticians who analyse the writing of the submitted analyses will monitor unblinded and/or subgrouped safety data, discuss results, and formulate and vote on recommendations for the study. Participating unblinded statisticians will provide guidance and answer statistical questions. The secretary of the DSMB will retrieve the analysis report and maintain it as a confidential document.

### Adverse event reporting and harms {22}

All AEs, including SAEs, which will be coded according to the definition of severe adverse events published by the FDA, will be carefully assessed for their severity, degree, and potential causality with the intervention or other possible treatments. Once an SAE occurs, the study will be terminated. SAEs will be reported to the institutional academic and ethics committee within 24 h and be managed by the attending physician according to standard medical practice, and we will continue to follow up with the patient until the symptoms resolve or the treatment is discontinued. The project will cover the cost of any adverse reactions related to the use of azvudine.

### Frequency and plans for auditing trial conduct {23}

The monitor will review the trial conduct monthly, including checking the original data of participants and counting the biological samples. The process will be independent of the researchers and the sponsor.

### Plans for communicating important protocol amendments to relevant parties (e.g. trial participants, ethical committees) {25}

In the event of any proposed changes to the protocol, these changes will be thoroughly documented in protocol amendments. These amendments will be submitted for approval to the ethics committee and regulatory agencies before implementation.

### Dissemination plans {31a}

The trial data will be publicly available for publication in international peer-reviewed journals, regardless of whether the results are positive or negative.

## Discussion

This is a double-blind, randomized, placebo-controlled, superiority, phase III clinical trial designed to evaluate the efficacy of azvudine in preventing mild-to-moderate COVID-19 patients who have the potential to develop severe COVID-19 from progressing to critical illness and death.

Since the beginning of the COVID-19 pandemic in 2019, it has afflicted at least 700 million people worldwide, and nearly 7 million human deaths have been caused by COVID-19 (http://covid19.who.int) [1]. Although evidence shows that the pulmonary pathogenicity of the variant Omicron strain has significantly weakened, the severity and mortality rates of elderly patients and those with underlying diseases are still high [23, 24]. SARS-CoV-2 will coexist with humans for a long time, and patients with underlying diseases still face a high risk of severe COVID-19 and death [25].

The WHO and China both have issued diagnosis and treatment guidelines for COVID-19 infection, in which antiviral treatment including Paxlovid, molnupiravir, and remdesivir targeting COVID-19 is the cornerstone [3, 4]. However, antiviral drugs such as Paxlovid are not suitable for some patients due to complicated drug interactions [26]. In addition, the high cost limits the wide use of such drugs in developing countries. Therefore, affordable, safe, and effective antivirals are in great demand.

RNA synthesis is an essential step in the replication lifecycle of coronaviruses and typically uses natural nucleosides from host cells as substrates for successful replication [27]. Nucleoside and nucleotide analogue inhibitors are synthetic molecules similar to purines and pyrimidines that may be mistakenly incorporated during virus replication, thereby terminating the synthesis of viral RNA chains or causing mutations that result in loss of viral viability or preventing viral replication and transmission [28, 29]. Nucleoside analogues have been successfully used to treat viral DNA and RNA infections, such as HIV, hepatitis B virus, and several respiratory viruses, such as influenza [30–34]. Azvudine, as a nucleoside analogue, has been developed for the treatment of HIV, and its clinical safety and effectiveness have been proven in HIV-positive patients [35].

Preliminary prospective studies regarding the treatment of azvudine in COVID-19 were carried out, and data on efficacy and safety were collected. For instance, a pilot randomized, open-label, controlled study accomplished in 2020 found that azvudine may shorten the time to nucleic acid-negative conversion in 10 patients with mild COVID-19 [36]. Another randomized clinical trial (RCT) was performed to evaluate the efficacy of azvudine for patients with mild COVID-19, which confirmed that azvudine was effective in reducing the viral load [20]. However, these RCT studies were small and did not report the efficacy of azvudine on mortality in COVID-19 patients.

Several single-centre retrospective studies reported the rate of disease progression and mortality after azvudine treatment in COVID-19 patients. They matched the demographics and disease severity of patients with COVID-19 through propensity score analysis and found that azvudine reduced the rate of disease progression, as well as the rate of COVID‐19‐related hospitalization. A retrospective study of mild-to-moderate COVID-19 patients showed that azvudine reduced the rate of disease progression and the hospitalization rate. Another study also showed that azvudine reduced the risk of disease progression in COVID-19 patients with preexisting conditions [37, 38]. However, inconsistent results are found among different studies regarding the impact of azvudine on all-cause mortality [17, 37–39]. Nonetheless, there are promising results showing that there was no difference in mortality between COVID-19 patients using Paxlovid and those using azvudine, which was a consistent finding in two retrospective cohort studies [40, 41].

In general, patients have a good tolerance to azvudine, and this drug may reduce the risk of critical illness and death in patients who are at risk of potentially progressing to critical COVID-19 infection.

As a new small-molecule antiviral drug, azvudine has the advantages of low price and less drug interactions compared with Paxlovid, an anti-COVID-19 drug used worldwide. However, due to insufficient research data, there is currently no multicentre, randomized clinical trial to prove the efficacy of this drug based on the endpoints of severe illness and death. The published research on the prospective cohort study of azvudine is limited to the impact on viral load, while research data support is still lacking for its impact on the critical disease rate and mortality of COVID-19, which clinicians truly care about.

In conclusion, a well-designed, randomized double-blind placebo-controlled study to assess the efficacy of azvudine in symptomatic adult COVID-19 participants who are at increased risk of progressing to critical illness is urgently needed. Our study will attempt to evaluate the difference in efficacy between azvudine antiviral treatment and placebo and to answer the important clinical question of the impact of azvudine on the critical illness rate and mortality of COVID-19.

## Trial status

The version of this protocol is 1.5 (date: May 18, 2023). Recruitment began in January 2023 and is estimated to be completed by January 2024. Currently, the trial is in the process of recruiting patients.

### Supplementary Information

**Additional file 1.** CONSORT flow chart of the progress of the study.

**Additional file 2. **SPIRIT Checklist for Trials.

**Additional file 3.** Consent form-V1.6–2023.07.17.

**Additional file 4.** List of study sites.

## Data Availability

The datasets used and/or analysed during the current study are available from the corresponding author upon reasonable request.

## Abbreviations

AE: Adverse event
ALT: Alanine transaminase
AST: Aspartate transaminase
COVID-19: Coronavirus disease 2019
DSMB: Data and Safety Monitoring Board
CRF: Case report form
eCRF: Electronic case report form
FAS: Full analysis set
FiO2: Fraction of inspired oxygen
IRT: Interactive response technology
IDMC: Independent data monitoring committee
mITT: Modified intention-to-treat
Paxlovid: Nirmatrelvir-ritonavir
PPS: Per-protocol set
RCT: Randomized clinical trial
RdRp: RNA-dependent RNA polymerase
SAE: Severe adverse event
SS: Safety set
SARS-CoV-2: Severe acute respiratory syndrome coronavirus 2
SpO_2_: Oxygen saturation
ULN: Upper limit of normal
WHO: World Health Organization
