# Supplementary figures and images for: Efficacy and safety of azvudine in symptomatic adult COVID-19 participants who are at increased risk of progressing to critical illness: a study protocol for a multicentre randomized double-blind placebo-controlled phase III trial
Source: Trials. 2024 Jan 22;25:77. doi: 10.1186/s13063-024-07914-3 (PMC10804629; doi:10.1186/s13063-024-07914-3)

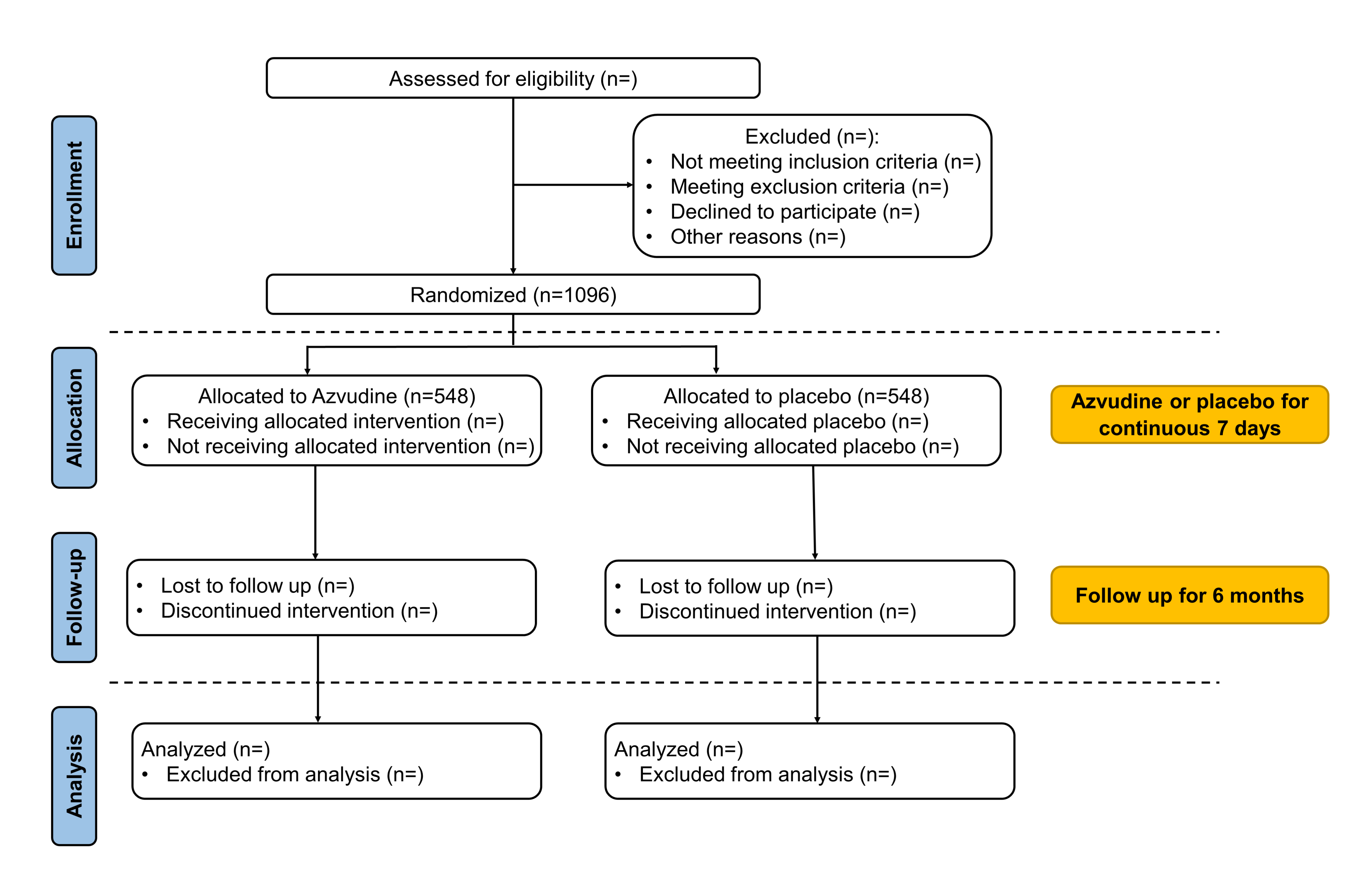

Supplement: Supplementary file 1 — Additional file 1. CONSORT flow chart of the progress of the study. [file 13063_2024_7914_MOESM1_ESM.tif]
