# Supplementary material for: Efficacy and safety of azvudine in symptomatic adult COVID-19 participants who are at increased risk of progressing to critical illness: a study protocol for a multicentre randomized double-blind placebo-controlled phase III trial
Source: Trials. 2024 Jan 22;25:77. doi: 10.1186/s13063-024-07914-3 (PMC10804629; doi:10.1186/s13063-024-07914-3)
Supplement: Supplementary file 3 — Additional file 3. Consent form-V1.6–2023.07.17. [file 13063_2024_7914_MOESM3_ESM.pdf]

关于阿兹夫定用于治疗有潜在进展为危重症新冠感染风险患者的多中心随机双盲安慰剂对照研究

知情同意书

申办单位：　北京协和医院

知情同意书•知情告知页

受试者须知

尊敬的受试者，您好！

我们邀请您参加一项《关于阿兹夫定用于治疗有潜在进展为危重症新冠感染风险患者的多中心随机双盲安慰剂对照研究》的临床试验。在您决定是否参加这项试验之前，请尽可能仔细阅读以下内容，它可以帮助您了解该项试验以及为何要进行这项试验、试验的程序和期限、参加试验后可能给您带来的益处、风险和不适。如果您愿意，您也可以和您的亲属、朋友一起讨论，帮助您做出决定。如果有不清楚的地方，您可以向您的负责医生咨询。一旦您所有的问题都被解答、您对有关这项研究的解释说明很满意、且您决定参加，您将被要求签署这份知情同意书。参加研究是您的自愿行为，您可以同意参加，也可以不同意。我们承诺，作为研究者，我们在这项临床研究中不存在利益冲突。

一、 您将服用什么药物？

本品为阿兹夫定片（FNC），英文名 Azvudine；商品名：阿兹夫定片，制剂规格：1mg/片。1 类新药阿兹夫定片是由河南师范大学常俊标校长发明，由河南真实生物科技有限公司拥有自主知识产权的新型核苷类逆转录酶抑制剂，对 HIV-1 有很好的抑制活性。本品目前已经批准上市。

FNC 经体外活性研究数据已证明具有很好的抗新型冠状病毒的作用，且经过我国、巴西和俄罗斯三个国家开展的临床试验，也证实了 FNC 对救治新冠感染患者的一定疗效，如能加快病毒转阴时间、CT 恢复时间更短等。2022 年国家药监局附条件批准 FNC 增加治疗新型冠状病毒肺炎适应证，国家卫健委将该药纳入《新型冠状病毒感染诊疗方案（第十版）》。目前现有的抗病毒药种类少，费用昂贵，难以获得。鉴于目前新型冠状病毒感染在全球、特别是我国的扩展迅速，已成为全世界人类健康的重大威胁，一旦出现重症和危重症，病死率高，因此抗病毒治疗需要关口前移，避免危重症患者产生。经北京协和医院伦理委员会批准，拟开展研究者发起的 FNC 治疗有潜在进展为危重症新冠感染风险成年患者的有效性和安全性临床研究。

二、本试验的目的是什么？

研究目的：评价阿兹夫定片治疗有潜在进展为危重症新冠感染风险成年患者的有效性和安全性

三、哪些人可以参与本试验？

1、入选标准

您应全部符合以下标准才能进入试验：

（1） 年龄 18 周岁以上（含界值）；

（2） 症状 5 天内的新冠感染，且入选前 5 天内的新冠核酸或抗原阳性；

（3） 入选时患者存在至少一种与新冠感染相关的症状；

（4） 育龄女性患者需要有效避孕（入组时尿妊娠阴性）；

（5） 至少存在一种新冠重症感染的高风险因素：

① 年龄≥65 岁；

② BMI >30；

③ 发热（体温≥38℃）≥3 天；

④ 现正吸烟者（30 天内仍在吸烟，且至少有 100 支以上烟龄）；

⑤ 免疫抑制性疾病，包括并不限于：骨髓抑制或器官移植或原发性免疫缺陷病；长期使用免疫抑制药物(最近 30 天之内，以泼尼松为例≥20 mg/d 使用至少 14 天)；接受生物制剂治疗（如英夫利昔单抗等）；使用免疫调节剂（包括并不限于甲氨蝶呤、硫唑嘌呤、环磷酰胺等）；恶性肿瘤 90 天之内接受过放化疗（其中胸部放疗应在 6 个月以上）；

⑥慢性肺病（如每天需要治疗的哮喘，支气管扩张、COPD、肺动脉高压、OSAS、肺间质病变等）；

⑦高血压；

⑧心血管病（既往曾被诊断为心肌梗死或脑卒中、TIA 发作、心功能不全、需要硝酸酯治疗的心绞痛、CABG、PCI 术后、颈动脉内膜切除术和主动脉旁路术术后等）；

⑨1 型或 2 型糖尿病；

⑩神经发育异常（如脑瘫、唐氏综合征）或其他遗传或代谢综合征和严重先天畸形；

⑪活动性肿瘤（不包括局限性皮肤癌）；

⑫未接种新冠疫苗且本次为首次感染新冠。

2、排除标准

您符合以下任何一项标准将不得进入本研究

（1） 已知或怀疑对阿兹夫定片的组成成分过敏；

（2） 根据当前试行最新版《新型冠状病毒感染诊疗方案》，确诊为重型或危重型新型冠状病毒感染患者（重型：1. 出现气促，RR≥30 次/分；2.静息状态下，吸空气时指氧饱和度≤93%；3.动脉血氧分压（PaO2）/吸氧浓度（FiO2）≤300mmHg；4. 临床症状进行性加重，肺部影像学显示 24～48 小时内病灶明显进展＞50%。危重型：1. 出现呼吸衰竭，且需要机械通气；2. 出现休克； 3. 合并其他器官功能衰竭需 ICU 监护治疗）；

（3） 合并严重的肝脏疾病（总胆红素（TBIL）≥2 倍正常值上限，丙氨酸氨基转移酶（ALT）、天门冬氨酸氨基转移酶（AST）≥3 倍正常值上限）；

（4） 合并有严重肾功能不全（肾小球滤过率≤60 mL/min/1.73 m^2）或正在接受连续性肾脏替代治疗、血液透析、腹膜透析者；

（5） 合并有吸收障碍综合征，或其它任何对胃肠道吸收产生影响的状况，需要静脉营养或不能口服药物的患者；

（6） 已知患有 HIV 感染；

（7） 出现糖尿病酮症或高渗状态者；

（8） 总中性粒细胞计数＜750 cells / L；

（9） 妊娠期、哺乳期妇女或在试验期间及结束 6 个月内有生育计划；

（10） 正在参加其他临床试验或正在使用试验性药物；

（11） 除新冠感染之外，还合并其他未控制的活动性感染（需病原学证实）；

（12） 研究者确定，研究开始前 7 天内需要住院和/或手术的任何共病，或研究开始前 30 天内被认为有生命危险的共病；

（13） 已接受或预计将接受恢复期新冠感染血浆；

（14） 本次新冠感染已经使用过明确有效的治疗新冠的抗病毒药物（包括但不限于奈玛特韦/利托那韦或莫诺拉韦，其中因非新冠原因应用糖皮质激素不适用于本条目）；

（15） 根据研究者的判断，具有不适合参加本试验的其它情况。

四、本试验有哪些流程及内容？

如果您符合入组条件，您将可能被分配至试验组或对照组，需按照方案要求接受研究药物每日一次，每次 5mg 口服治疗，给药 7 天。在这期间需要您接受 3 次新冠核酸定期检查。

给药方法：口服给药，每日服药一次，每次 5mg。给药方法详见下表：

组别	研究药物	给药剂量
试验组	FNC	1mg×1 片，5 片/日
对照组	安慰剂	1mg×1 片，5 片/日

研究流程：

（1）筛选期

作为筛选的一部分，您的负责医生将记录您的人口学信息（民族、年龄、性别、身高、体重）和病史和手术史（包括目前和既往患有的疾病和接受的治疗）、过敏史、用药史、吸烟史、酗酒史、药物滥用史以及临床试验参加史。

您需进行如下检查：

- 主诉症状；
- 生命体征；
- 尿妊娠（育龄期女性受试者）；
- 血常规；
- 血生化；
- 血氧检查。

您的负责医生将根据入选和排除标准评估您是否符合参加本试验的条件，若受试者符合全部入选标准且不符合任一排除标准即可入组本试验。

（2）治疗期（d1~d7）

如果您符合本试验的入选条件，您将接受给药最多至 7 天，或如果您病情加重，可以选择继续服用 FNC 至转病毒核酸阴，也可以选择停用。

由于本研究是随机双盲的临床研究，受试者将会被分为两组：试验组和对照组进入两组的比例是 1:1，分组的方式是随机的（就像抽签一样），您和研究者都不能事先选择参加到哪个组。您有一半的可能性接受 FNC 药物，也有可能一半的可能性获得安慰剂，安慰剂为 FNC 药物的辅料（不含活性成分），由 FNC 的生产厂家：河南真实生物科技有限公司提供，不具有任何危害健康的成分。您和医生或家人，在研究完全结束前，均不知您是否吃到的是药物或安慰剂。

研究期间进行常规访视，用药第 1 天至用药第 7 天您需每天用药，在用药后第 1 个月您需进行复查，您的负责医师将询问您用药后的感觉及临床症状并进行一系列检查，其中用药第 1 天至用药第 28 天每天都需要自我报告症状（如果症状消失 3 天以上，可以停止报告），用药第 1 天、第 3 天、第 7 天、第 14 天需要进行病毒核酸定性检测，并进行生命体征监测和必要的采血、胸部影像学检查（第 7 天、第 14 天及第 28 天，如果患者一般情况改善，本次影像学检查可以无。如果研究者判断患者不需要进行 CT，但患者希望开具，本部分费用由患者本人支付），以确定病情和用药的安全性。

用药第 1 天（d1，现场访视）

您需要进行以下检查：

- 主诉症状；
- 生命体征；
- 咽拭子新冠核酸/抗原检查；
- 肺部 DR/CT；
- 血常规；
- 铁蛋白（本次检查为推荐检查项目，非本研究不需检查项目，可由研究者与受试者协商是否进行此项检查）；
- 白介素-6（本次检查为推荐检查项目，非本研究不需检查项目，可由研究者与受试者协商是否进行此项检查）；
- hsCRP；
- 血生化；
- 凝血功能；
- 心脏相关检查（肌钙蛋白 I（cTnI）、N 末端前体脑利钠肽（NTproBNP））；

新冠病毒载量检测。如果您已有本研究中心出具的 d1 前 48 小时内的某项检验结果或

者 72 小时内的胸部影像学结果，那么该项目可不再重复检验/检查。

用药第 3 天（d3，现场访视）

您需要进行如下检查：

- 主诉症状；
- 生命体征；
- 咽拭子新冠核酸/抗原检查；
- 新冠病毒载量检测；
- 血常规；
- hsCRP；
- 血生化。

用药第 4~6 天（d4~d6）

在此期间，需记录你的主诉症状。

用药第 7 天（d7，现场访视）

您需要进行以下检查：

- 主诉症状；
- 生命体征；
- 咽拭子新冠核酸/抗原检查；
- 肺部 DR/CT（如果您的一般情况改善，本次影像学检查可以无。如果研究者判断您不需要进行 CT，但您希望开具，本部分费用由您本人支付）；
- 血常规；
- hsCRP；
- 血生化；
- 凝血功能；
- 心脏相关检查（肌钙蛋白 I（cTnI）、N 末端前体脑利钠肽（NTproBNP））；
- 新冠病毒载量检测。

治疗期间需详细记录合并用药和不良事件发生情况。

（3）研究完成

进行 28 天的健康监测，避免劳累和过度运动；

在治疗开始后第 2 周和第 4 周进行复诊，第 4 周的检查项可根据研究者要求进行调整；

第 8~13 天（d8~d13）

在此期间，需记录你的主诉症状。

第 14 天（d14，现场访视）

- 主诉症状；
- 生命体征；

- 咽拭子新冠核酸/抗原检查（如果此前新冠核酸/抗原已转阴，本次核酸/抗原检查可以无）；
- 尿妊娠（育龄期女性受试者）；
- 肺部 DR/CT（如果您的一般情况改善，本次影像学检查可以无。如果研究者判断您不需要进行 CT，但您希望开具，本部分费用由您本人支付）；
- 血常规；
- hsCRP；
- 血生化；
- 新冠病毒载量检测。

第 28 天（d28）

- 在此期间，需记录你的主诉症状。
- 肺部 DR/CT（如果您的一般情况改善，本次影像学检查可以无。如果研究者判断患您不需要进行 CT，但您希望开具，本部分费用由您本人支付）。

从用药 1 月内出现发热和/或呼吸道症状等，须立即向研究者报告。

若您在试验期间出现不良事件，根据研究者判断，可回院/在其他医院进行检查随访，直至有转归结果，或电话随访。

为了了解 FNC 对您的潜在影响，我们邀请您研究结束后的 3 个月和半年再次进行当面或电话随访。并进行以下检查：

第 3 个月（3M）、第 6 个月（6M）

在此时，需记录你的主诉症状。

五、本试验您需要参与多长时间？

本试验共 7 次访视（其中 4 次现场访视，3 次现场访视或电话访视），共预计需要您持续参与 14 天，但会在 28 天、3 个月和 6 个月进行 3 次电话或现场访视。

六、本试验您的义务是什么？

您应当了解作为这项研究的参与者，您有一些相应的义务。您有责任向您的负责医生报告在试验过程中您身体和精神方面的任何改变，无论您认为这种改变是否与这项研究有关。

开始研究前请您务必告知我们您目前正在服用和在研究期间将要服用的任何其他药物，包括任何处方药物、非常规维生素和中草药等的非处方药物。在研究期间如果您因为任何疾病需要到医院就诊或住院治疗，请告知医生您正在参加此项试验，以便医生更好地了解您的情况。一旦发生上述情况，请尽快通知您的负责医生。

七、参加本项研究可能的受益

您和社会将可能从本项研究中受益。但我们不能保证肯定受益，需获得具体研究结果数据，且经过科学统计分析后才能具体判断。您的获益可能表现为您的疾病在本试验得到有效治疗；社会的获益可能表现为本项研究帮助开发出一种新的治疗方法，以用于患有相

似病情的其他病人。这些信息将有益于帮助将来的患者。

八、研究治疗的花费是多少？

试验期间您的试验用药物和核酸检测由研究者免费提供，入组首次的检查是为了评估您的疾病严重度，花费由您自己承担（均为医保范畴的检查）。但是观察期间 D3，D7 和 D14 的血常规、血生化与药物副作用相关的检查（每次约 140 元，共计 420 元左右）和您返院随访的交通费，由研究者承担。如果医生判断您由于新冠本身的病情变化需要增加其他检查，所发生的费用由您自行承担。

九、参加本项研究可能的风险及保护措施

任何药物都有可能给您带来不适。

本试验药物单次给药的安全药理研究结果显示：在犬和小鼠为动物模型中，FNC 对小鼠的交配、生育力及胚胎发育未出现异常，FNC 仅可少量通过血乳屏障，经乳汁排出。

I 期临床试验中共入组 40 例未接受治疗的 HIV 感染者进行单次给药，未出现严重不良反应，不良反应主要为发热、头痛、头晕、恶心、呕吐、腹泻，严重程度均为 1 级，除了 2 例受试者发热采用扑热息痛（1 片/1 次，2 次，1 天）和泰诺（1 片）治疗外，均未采取任何措施自行恢复；共入组 16 例未接受治疗的 HIV 感染者进行多次给药，2mg 和 4mg 分别有 3 例和 2 例受试者发生 5 次和 2 次不良事件（AE），仅 4mg 组 1 例 1 次 AE 判定为与研究药物可能有关，严重程度II级，表现为给药第 4 天发生中性粒细胞绝对值降低，未给与治疗，出组检查时已恢复消失/复常，其余 6 次 AE 严重程度均为 1 级，5 次 AE 判定为与研究药物可能无关，1 次 AE 肯定有关。

II期临床试验共计入组 172 例受试者，其中 2 例受试者撤回知情同意书，15 例受试者因耐药、依从性不佳、自愿更换为自费药物、不良事件等原因退出研究，155 例完成 48 周访视。总体来说，受试者耐受性良好，对治疗方案的依从性高，发生率最高的不良反应为头晕，其次为丙氨酸氨基转移酶升高、γ-谷氨酰转移酶升高、天门冬氨酸氨基转移酶升高、血尿酸升高。

除此之外，您应当了解，在创新药物研究中，可能出现一些不可预知的不良反应，或者在研究中也可能出现一些与试验药物无关但会对您健康造成不良影响的情况，如采血过程可能因个人耐受情况导致短暂头晕等不适。这是目前我们无法预知的。

试验期间，若您出现任何不适，请您及时告知研究者，研究者将根据您的具体情况给予您对症治疗来确保您的安全。

十、妊娠和哺乳：

本品对妊娠或者哺乳的风险尚不明确。如果您已怀孕或正处于哺乳期，您不能参加本试验。如果您在研究期间受孕，将此信息告知您的医生是非常重要的。在入选本试验前，会进行一次妊娠或两次试验以确保您没有怀孕。在本试验期间，您必须使用有效的避孕措施，适当的避孕方法包括宫内节育器、使用带杀精子剂的子宫帽或避孕套等非药物避孕。如您是男

性，建议您在参加本试验期间采取有效的避孕措施，确保在研究期间伴侣不受孕。您的避孕时间应当持续到至少为停药后 6 个月。

试验期间，如果您的避孕措施出现任何差错，请立即通知您的负责医生，由您的负责医生和产科医生共同做出判定，您的负责医生将跟踪您/您伴侣的妊娠状态直至结果明确，包括流产或自动终止妊娠，分娩细节，是否存在任何出生缺陷，或先天畸形，或母亲的和新生儿的并发症。

十一、参加研究可能获得的补偿

我们将为您购买研究保险，若您参加本研究发生研究相关损害，将按照您的具体损伤情况进行补偿。对参与研究的受试者，给予一定的交通费补偿，将按照您完成第 1 天、第 3 天、第 7 天、第 14 天和第 28 天的访视检查的情况，您将获得每次 200 元的交通补贴。

十二、您可以自愿选择参加研究和中途退出研究

如果您选择参加本项研究，我们希望您能够坚持完成全部研究过程。

如果您在研究过程中的任何时间退出本试验，这都不会影响您和医生的关系，也不会影响您的医疗或有其他方面利益的损失，您的负责医生将安排您继续接受常规的医疗护理。

十三、您个人信息的保密

如果您签署知情同意书同意参加此项试验，意味着您同意研究者的指定人员或代表、国家药品监督管理局及伦理委员会可以直接查阅您的病历记录。查阅的目的是确保准确收集试验资料，保证试验严格按照研究方案进行。上述人员将确保您的个人资料保密，除非在极少数情况下，根据法律或司法程序必须向其他人员透露。试验的相关资料将有可能被用于科学研究或文章发表，但不会包括您的名字、身份、电话号码等私人信息。

除本试验以外，有可能在今后的其他研究中会再次利用您的医疗记录，同样不会披露您的个人身份。

十四、如果您有问题或困难该与谁联系？

伦理委员会已经审查通过该研究，如果您有与自身权利/权益相关的任何问题，或者您想反映参与本研究过程中遭遇的困难、不满和忧虑，请联系北京协和医院临床研究伦理委员会，联系人：李佳月，联系电话：010-69156874。

如果您在研究过程中出现任何不适或对该研究存在任何疑问，请联系研究单位北京协和医院呼吸与危重症医学科联系，联系人：武老师，电话：13581521503。

现在该做什么

是否参加本项研究由您自己决定。您可以和您家人或者朋友讨论后再做出决定。

在您做出参加研究的决定前，请尽可能向您的医生询问有关问题，直至您对本项研究完全理解。

感谢您阅读以上材料。如果您决定参加本项研究，请您在本知情同意书上签名。您将得到一份已签署知情同意书的副本，请妥善保存。

知情同意书·同意签字页

研究题目：关于阿兹夫定用于治疗有潜在进展为危重症新冠感染风险患者的多中心随机双盲安慰剂对照研究

申办单位：北京协和医院

伦理委员会批准号：

患者同意声明

我已经阅读和理解了所有提供给我的参加此项临床试验的相关信息，并且有时间和机会提出问题。所有我提出的问题都已经得到了满意的答复。我自愿参加此项研究。我保证我所提供的所有个人资料，包括我的病史，都是真实和尽可能准确的。我同意本研究使用我的医疗资料。

我知道我可以获得这份已签署的知情同意书的副本。

最后，我决定同意参加本项研究，并保证尽量遵从医嘱。

受试者（正楷）：

受试者签名：

联系电话：

日期：　　年　　月　　日　　时　　分

医生声明

我确认已向患者解释了本试验的详细情况，包括其权利以及可能的受益和风险，并给其一份签署过的知情同意书副本。

研究者（正楷）：

研究者签名：

联系电话：

日期：　　年　　月　　日　　时　　分
